# Whitworth Chronic Hospital, Dublin

**Published:** 1831-09

**Authors:** 


### WHITWORTH CHRONIC HOSPITAL, DUBLIN.

### *Case of Melanosis.* By John Crampton, m.d.*

Daniel Browne, aged thirty-four, a weaver, was admitted into the Whitworth Chronic Hospital, on the 28th June, 1828. He laboured under ascites to a great extent, the abdomen being unusually prominent, and his disorder was of six years standing. Fluctuation was distinctly perceptible, but a part of the distention evidently arose from solid enlargement of the liver: the margin of this viscus could be traced extending transversely into the left hypochondre, which it filled completely, and downwards to within an inch of the spine of the ilium on both sides, and fully above two inches below the umbilicus. Several tumors were felt on its surface, of which the patient himself was sensible. He suffered no pain, excepting the distress occasioned by the incumbrance of the tumid organ, and the distention of the abdominal parietes; lies on either side, but expresses himself easier when he sits up; his breathing is laborious, but the thoracic organs appear sound, as was evinced both by percussion and auscultation; his bowels were confined, except when relieved by medicines; passes very little urine, and that high coloured; has been very intemperate in drinking spirits to excess.

The case I considered to be one of tuberculated liver enlarged to a great extent, of long standing, with consecutive dropsical effusion, and no expectation was entertained as to a chance of recovery. Remedies were, of course, prescribed to relieve his bowels and promote the secretion of urine, but their effects were only temporary.

On the 26th July, he was in great distress from the enormous distention of his abdomen, and he earnestly requested relief by

* Dublin Medical Transactions.

tapping: his entreaties were complied with, the paracentesis was performed, and four gallons of fluid were removed, with instantaneous relief, and he spent the night comfortably; but the following day he complained of soreness inside the belly. At this time the enlarged liver, with its tumors, could be felt still more distinctly, in consequence of the abstraction of the fluid.

From this period until that of his death, which took place a few days after, he gradually sunk, the pain and soreness in the abdomen increasing, unable to derive any benefit from the treatment adopted. The immediate cause of his death appeared to be inflammation of the peritoneum: this, as frequently happens after tapping, commenced at the puncture, and spread to a considerable extent over that membrane.

His body was examined four hours after death.

External appearances: Considerable emaciation; evident fluctuation in the abdomen; margin of the liver can be traced as described in the history of the case.

Thorax: The pleuræ were studded with black tumors, each about the size of a pea: when cut into, they resembled coagulated blood; the lungs and bronchia were perfectly sound, and the heart natural.

Abdomen: On opening this cavity, upwards of a gallon of fluid was discharged. The surface of the contents of the stomach was covered with immense flakes of lymph, which appeared quite recently formed: this adhered everywhere in bands, and glued the intestines together and to the liver. The peritoneum was of a brownish colour, covered with a false membrane, which was easily scraped off with the knife. The mucous membrane of the stomach was very vascular; that of the intestines did not present any thing very remarkable; their serous covering, however, was much thickened.

Nearly two thirds of the abdominal cavity were occupied by the liver: the right lobe extended up into the corresponding side of the thorax, compressing the lungs on that side to half their natural dimensions. This immense viscus stretched across and occupied the whole epigastrium, and reached fully two inches below the umbilicus, to within an inch of the spine of the ilium on both sides. On its surface it was thickly studded with round black tubercles, varying in size from that of a small plumb to that of a middle-sized apple. When cut, these tubercles presented a soft pulpy matter, easily broken down, and resembling Indian ink in colour, staining every thing which came in contact with it. The interior of the liver was also studded with these black tubercles, so that, when cut, the surface resembled a slice of plum pudding. The liver weighed nineteen pounds; its circumference was equal to three feet eight inches. Transverse diameter of the under surface, fifteen inches; antero-posterior diameter along the horizontal fissure, ten inches; that of the right lobe, twelve and a quarter inches; that of the left lobe, eleven and a half inches.

The spleen was healthy in structure, but smaller than natural, having been compressed by the liver. The left kidney contained several calculi in separate cysts, some black, like coal, others of the uric-acid kind: two similar ones were seen in the right kidney. These calculous concretions were found in the tubular portions of the kidney.

A drawing has been taken of a sliced portion of the liver, and some pieces of that altered viscus have been preserved in spirit.

Melanosis is not a common occurrence to those who are in the habit of examining bodies, nor is it accompanied by any symptoms which would seem to form a diagnosis between it and other changes of structure, which we more frequently meet with in the lungs, liver, and other important organs. The older writers have described some cases of it; but they confounded this morbid production with scirrhus, cancer, or carcinoma, and in some instances with the black matter found in the healthy lungs of elderly people.

The name Melanosis was first given it by Laennec, the talented author of the "Auscultation Mediate:" he described this affection in an unpublished paper, which he presented to the Ecole de Medecine in 1806. He afterwards, in his book published in 1819, considered it a *tissu accidentel;* that it was first a solid texture, and afterwards became softened and was converted into cavities, like other tubercular formations; but that its presence was not denoted by any peculiar symptoms. This opinion of Laennec has been since contested by later writers. Breschet gives some account of melanosis in Magendie's Journal for October 1821; there is also one by Drs. Cullen and Carson, in the first vol. of the Edinburgh Medico-Chir. Transactions. An excellent description of melanosis in different organs is published by Mr. Fawdington, London, 1826, where he exhibits all the varieties of the disease. A description likewise may be found in Mr. Wardrop's edition of Dr. Baillie's works; but Andral gives a more complete view of the subject in the Dict. des Sciences Medicales, and a still later one in his Precis d'Anatomie Pathologique, 1829. Andral objects to its being denominated a *tissu,* as it is completely inorganic, and may exist in different forms: first, in masses, and these masses either with or without cysts, derived from the cellular texture; secondly, infiltrated or diffused through textures, so as to be incorporated with another tissue; thirdly, spread out into solid strata or layers, especially on the serous membranes; and fourthly, in a liquid state. Examples of this kind may be seen on the mucous membrane of the intestines, where they have been observed covered with an inky liquid secretion after inflammatory affections, and in the cavity of the abdomen after chronic peritonitis. Immense melanose collections have been found in the abdomen of horses, according to the observations of Trousseau, Leblanc, and Dupuy.

In point of chemical analysis, the composition of melanose collections does not differ from that of the blood: albumen, fibrin, a carbonaceous matter, oxyd of iron, and the usual saline ingredients of that fluid, being found in both, according to the experiments of Messrs. Thenard, Clarion, Lassaigne, Barruel, and Foy. M. Barruel considers the colouring matter of the blood the furnishing material from whence the matter of melanosis proceeds. Thus far pathological anatomy, aided by chemistry, leads us as to the nature of these extraneous or accidental developements; but as to the mode of their formation, or the way in which this perverted secretion takes place, we have little to offer but conjecture.

